# Speech Impaired by Half Masks Used for the Respiratory Tract Protection

**DOI:** 10.3390/ijerph19127012

**Published:** 2022-06-08

**Authors:** Krzysztof Nowacki, Karolina Łakomy, Wojciech Marczak

**Affiliations:** 1 Department of Production Engineering, Faculty of Materials Engineering, Silesian University of Technology, Akademicka 2A Street, 44-100 Gliwice, Poland; karolina.lakomy@polsl.pl; 2 Faculty of Science and Technology, Jan Długosz University, Al. Armii Krajowej 13/15, 42-200 Częstochowa, Poland; w.marczak@ujd.edu.pl

**Keywords:** filtering half masks, suppression, voice, protective measures, speech intelligibility

## Abstract

Filtering half masks belong to the group of personal protective equipment in the work environment. They protect the respiratory tract but may hinder breath and suppress speech. The present work is focused on the attenuation of sound by the half masks known as “filtering facepieces”, FFPs, of various construction and filtration efficiency. Rather than study the perception of speech by humans, we used a generator of white noise and artificial speech to obtain objective characteristics of the attenuation. The generator speaker was either covered by an FFP or remained uncovered while a class 1 meter measured sound pressure levels in 1/3 octave bands with center frequencies 100–20 kHz at distances from 1 to 5 m from the speaker. All five FFPs suppressed acoustic waves from the octave bands with center frequencies of 1 kHz and higher, i.e., in the frequency range responsible for 80% of the perceived speech intelligibility, particularly in the 2 kHz-octave band. FFPs of higher filtration efficiency stronger attenuated the sound. Moreover, the FFPs changed the voice timbre because the attenuation depended on the wave frequency. The two combined factors can impede speech intelligibility.

## 1. Introduction

Airborne dust in the working environment is a health risk factor, e.g., in mining [1], wood and furniture [2,3], and construction industry, as well as in the welding and grinding [4] and biomass processing [5]. Half masks covering the nose, mouth, and chin are personal protective equipment against harmful physical and biological agents. A European standard EN 149:2001+A1:2009 specifies three classes of the half masks called filtering facepieces (FFP): FFP1, FFP2, and FFP3 [6]. These are mechanical filter respirators with various filter efficiency, equipped or not with inhalation or exhalation valves. Healthcare workers use FFPs as protection against bacteria and viruses, SARS-CoV-2 in particular [1,7]. World Health Organization recommends several types of masks for the public to reduce the transmission of viruses [8]. However, the recommendations are not entirely followed even in hospitals, although the situation in the COVID-19 wards is better than in others [9].

Although it improves safety, personal protective equipment may cause work to be uncomfortable. For this reason, workers sometimes do not obey the rules of personal protection in small and medium-sized enterprises in particular [3]. The respirators may cause difficulty in breathing due to clogging by dust [10]. Therefore, they are temporary rather than permanent protective equipment [11]. Indeed, obstructed breathing is a serious problem if a physical effort is required to perform work. Albeit the difference between resting energy expenditures measured for the subjects wearing and not wearing FFP2 masks proved statistically insignificant, the oxygen consumption and the carbon dioxide production were slightly higher with the filtering masks [12]. Choi et al. [13] found that the energy cost of a single inhalation varied depending on the type of a half mask in the range up to 10 mJ. That was about 7.1 mJ for half masks with a valve, and discomfort was rated 4.6 on a 6-point scale. Thus, the effort needed to inhale air contributes significantly to the comfort of wearing a half mask [13].

Another question is the impact of mask-wearing on speech clarity. Interest in this subject has increased as the masks as a countermeasure against the spread of COVID-19 have been adopted. Probably everyone noticed difficulty in verbal communication when wearing a mask. In particular, users of hearing aids and cochlear implants have to put more effort to understand the speech of mask-wearing persons [14]. However, Cohn et al. [15] reported that speakers with half-masks were more intelligible than those without the masks, provided they spoke clearly as to someone who might have trouble understanding the speaker. That was an exception rather than a rule. Masks worsened the intelligibility of casual and emotional speech [15]. Perception of speech in classrooms depended on the mask type used by the speaker apart from the speaker–listener distance [16]. An experiment involving twenty healthcare workers showed that speech recognition was decreased by 7% on average when speakers wore half-masks [17].

The reported works dealt mainly with speech perception by individuals. Apart from that, Oren et al. [18] studied the perception of the singing voices. Moreover, they analyzed changes in spectra of acoustic chirp signals caused by masks of several types: neck gaiter, disposable surgical mask, N95 mask (an equivalent of the FFP2 according to EN 149-2001 standard), and acoustic foam. In general, suppression and amplification of waves of particular frequencies depended on the mask type. N95 respirator suppressed acoustic waves of frequencies between 2 and 5 kHz and above 6 kHz. The authors concluded that the N95 respirator most strongly disrupted the auditory perceptual characteristics of the singing voice.

The SARS-CoV-2 pandemic spurred the development of filtering masks. The number of patents in March and April 2020 increased by about 100% compared with the period before the pandemic [19]. New designs improved the filtration efficiency and the wearing comfort [20,21,22]. However, we are not aware of the improved acoustic characteristics of the new masks despite the crucial role played by verbal communication in the life and work environment.

A majority of the studies reported in this brief review dealt with the perception of the human voice. Investigations of objective parameters, such as attenuation of sound waves by filtering masks, were scarce. We decided to fill that gap at least partially. Thus, we studied FFPs of various types using a calibrated source of the acoustic signal and sound level meter and analyzer.

## 2. Experimental

### 2.1. Research Material

**Figure 1:**
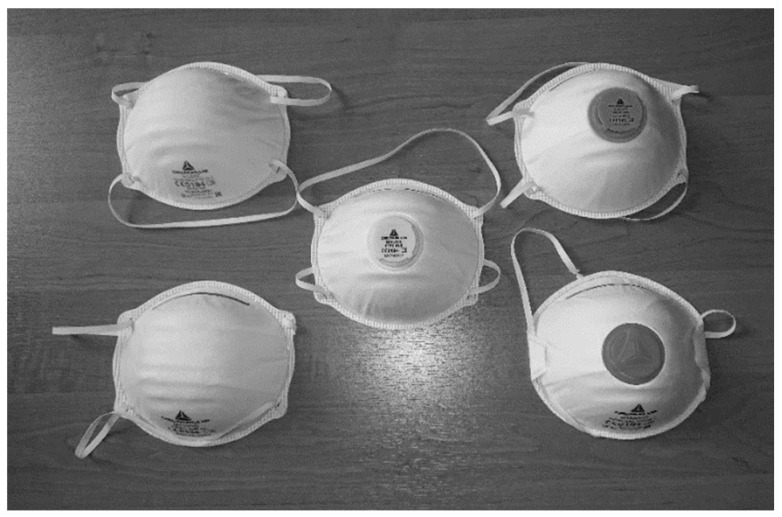
Filtering half masks—the research material. In columns: FFP1, FFP2, FFP1+, FFP2+, FFP3+.

We tested five convex-shaped disposable filtering half-masks of the FFP type, CE0194 certified according to EN 149:2001+A1:2009 standard [6] (Figure 1). All the masks were from one manufacturer, made of synthetic fiber, and equipped with an adjustable nose clip. The FFP1 and FFP2 were anti-dust half-masks without bactericidal inserts, while the FFP3 was a half-mask with such insert. The “+” sign in the FFP marking denoted a mask equipped with an exhalation valve for better breathing. Table 1 contains information about the filtering efficiency and applications of the studied half-masks.

### 2.2. Apparatus and Methodology

Svantek SVAN 979 class 1 sound and vibration analyzer compliant with the IEC 61672-1: 2013 standard [23], equipped with a GRAS 40AE 1/2″ microphone, was used in the measurements of sound pressures. Before and after each measurement series, the meter was checked with the class 1 Sound Calibrator SV36 according to the IEC 60942: 2017 standard [24]. Bedrock TalkBox BTB65 provided the acoustic signal. All apparatus used in the measurements had valid calibration certificates.

The measurements were carried out in a medium-sized laboratory room, about 11.7 m long, 6.2 m wide, and 3.2 m high. The reverberation time in the room was assessed for 0.3 s for the furniture and equipment arranged for this study, as that was for the standard classroom arrangement.

The microphone and TalkBox stood on tripods 1.6 m above the floor, which is the average mouth location of a standing adult human according to anthropometric data applied, e.g., in the C50 speech clarity measurements [25]. The speaker–microphone distance was from 1 to 5 m, and they were aligned using the built-in laser pointer of the TalkBox. SVAN 979 m analyzed and recorded the acoustic signal from the TalkBox in 1/3 octave-wide bands. The generated total sound pressure levels were 60 and 72 dB. Background level noise of ca. 28 dB was sufficiently small to neglect it. Sound pressure levels in 1/3 octave bands with center frequency from 100 Hz to 20 kHz were considered in further calculations.

**Figure 2:**
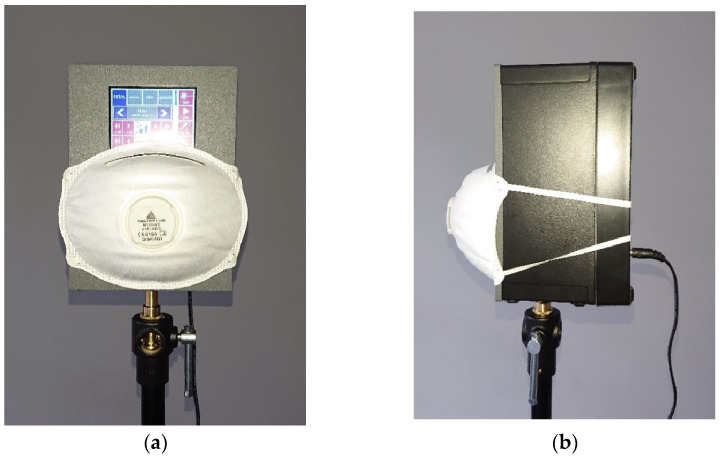
TalkBox with a tested half mask; (**a**)—front view, (**b**)—side view.

In the first series of measurements, the TalkBox emitted white noise, while in the second, it simulated human speech defined by IEC 60268-16:2020 standard [26]. The measurements were carried out for the TalkBox speaker uncovered and covered by each of the five FFPs. Figure 2 shows the TalkBox with a tested mask on the speaker. The masks fully covered the speaker. What may seem to be a gap between the box and the mask is a part of the box made of grey plastic.

## 3. Results

### 3.1. White Noise

In this measurement series, TalkBox emitted white noise and its immission was recorded by the SVAN 979.

Each measurement of the sound pressure levels lasted 60 s, divided into six 10 s-long intervals. In this manner, six sets of the sound pressure level values in 1/3 octave-wide frequency bands, *L_f_*_,10s_, were recorded for each experimental arrangement. The latter included: the TalkBox speaker (covered by one of the five FFPs or without cover), the speaker–microphone distance (*d* = 1 m or 5 m), and the white noise pressure level (*L_wn_* = 60 or 72 dB). The raw *L_f_*_,10s_ results are in the attached Supplementary Files: “White noise 1 m” and “White noise 5 m”. Since the *L_f_*_,10s_ values for the given experimental arrangement and center frequency *f* were equal within the uncertainty range, they were averaged for the 60 s-long measurement time. Finally, the *L_f_*_,60s_ values were calculated for octave-wide bands to match the frequency bands of the ANSI speech spectrum [27].

Attenuation of sound by the FFP in the octave band of center frequency *f* is the following difference between the respective *L_f_*_,60s_ values:

Δ*L_f_*_,60s_ = *L_f_*_,60s_ (no FFP) − *L_f_*_,60s_ (with the FFP). (1)

Four sets of Δ*L_f_*_,60s_ values were obtained for each FFP studied from Equation (1). They are reported in Table 2, together with respective average values calculated according to the additivity rule for the squared sound pressures. The averaging was possible because the particular Δ*L_f_*_,60s_(*d*,*L_wn_*) values for given *f* were equal within the measurement uncertainty limits for the class 1 m. The latter is ±1.1 dB for *f* = 1 kHz and is higher for other frequencies [23].

**Figure 3:**
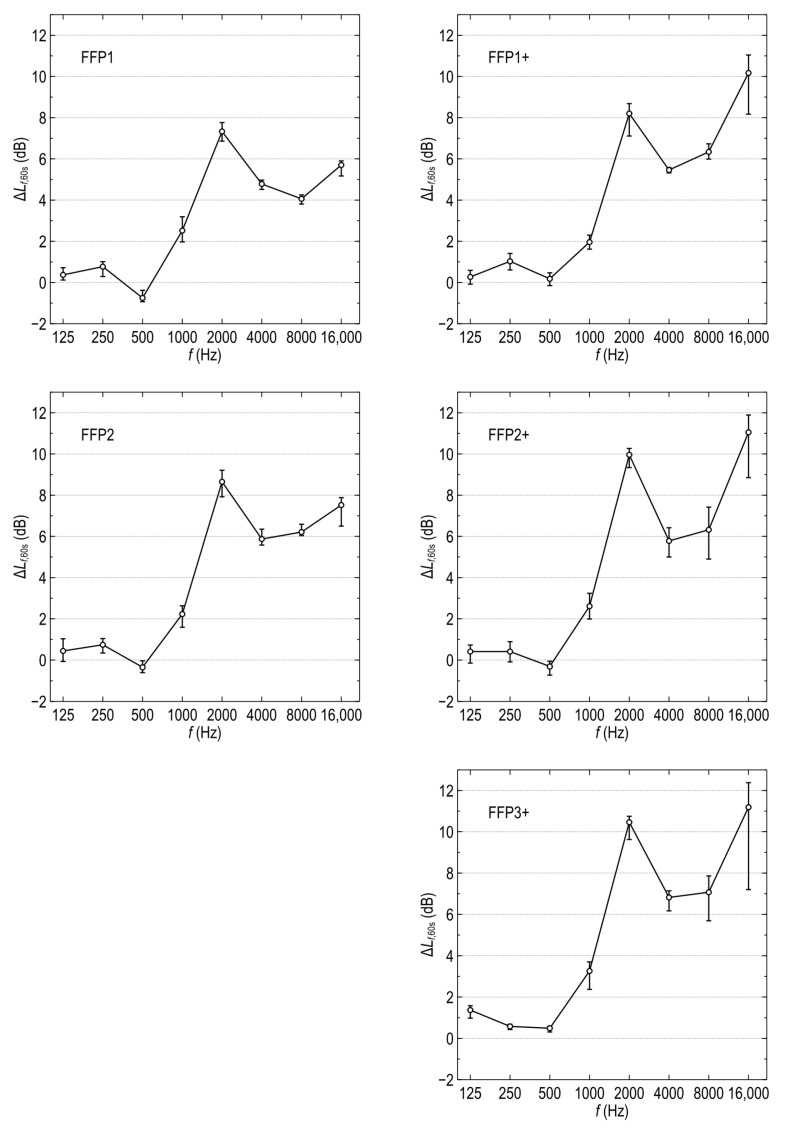
Attenuation of sound by the five FFPs in octave bands with center frequencies *f*. Points—averaged values Δ*L_f_*_,60s_ (cf. Table 2), whiskers—minimum-maximum range. Lines are guides for the eye only. Plus sign in the FFP symbol denotes a mask with an exhalation valve.

The Δ*L_f_*_,60s_ are plotted in Figure 3. Note that small negative values of Δ*L_f_*_,60s_ are within the measurement uncertainty limits and do not prove the signal amplification. 

### 3.2. Simulated Speech

The primary goal of the second experiment was to collect data for a comparison of the speech disruption predicted from the FFPs attenuation characteristics with the results of direct measurements. The measurements regime was similar to the previous one, except that TalkBox emitted simulated human speech defined by IEC 60268-16:2020 standard [26] rather than white noise.

The speaker–microphone distance *d* was 1, 2, 3, 4, or 5 m, the emitted maximum sound pressure level *L*_hs_ was 72 dB, and the averaging time of the measured sound pressure levels was 1 s, while the total measurement time was 10 s in each run. The SVAN 979 meter analyzed the acoustic signal and recorded the sound pressure levels in 1/3 octave bands.

Three samples of each FFP type were tested for within-subject variability. The Shapiro–Wilk test evidenced that distributions of the acoustic pressures in 1/3 octave-wide frequency bands differed from the normal distribution at the 5% level of significance. However, the distributions for each FFP type did not show statistically significant differences in the Kruskal–Wallis ANOVA test, and respective median values of the sound pressure levels also did not.

For consistency with the FFPs attenuation characteristics, the 1/3 octave sound pressure levels were summed up in each octave-wide band with center frequencies from 125 Hz to 16 kHz. In this manner, 120 experimental time series of the *L_f_*_,1s_ for each FFP were obtained. Further, they could be compared with the *L_f_*_,1s_ series calculated from the attenuation characteristics of the FFPs, Δ*L_f_*_,60s_ reported in Table 2, according to the following formula:

*L_f_*_,1s_ (with the FFP) = *L_f_*_,1s_ (no FFP) − Δ*L_f_*_,60s_. (2)

**Figure 4:**
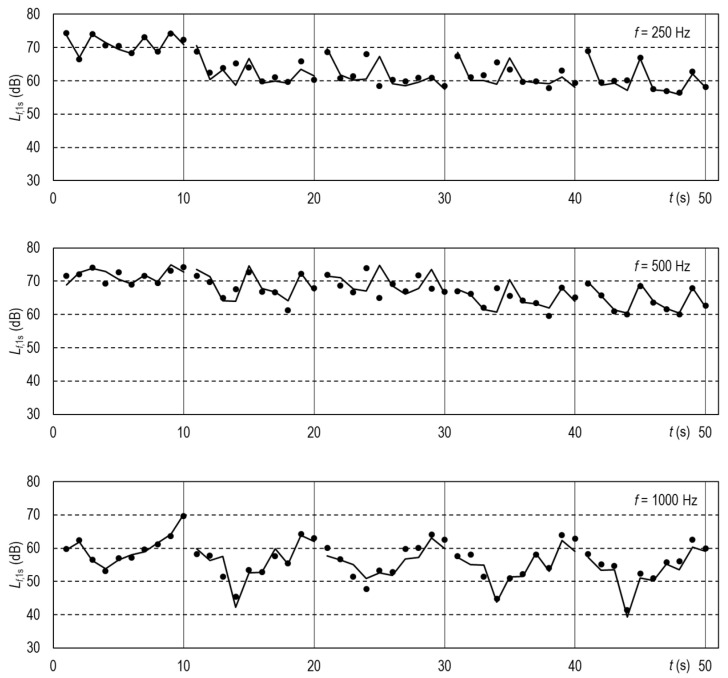

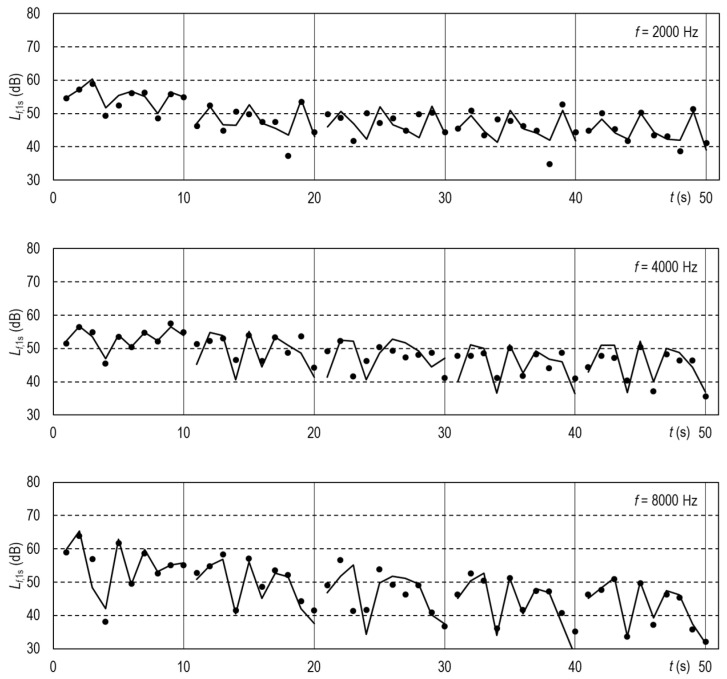
Time series of the sound pressure levels for the FFP1 in octave bands averaged for 1 s-long intervals. Five 10 s-long samples of the simulated human speech emitted by the TalkBox and recorded at distances (from left to right): 1 m, 2 m, 3 m, 4 m, and 5 m. Points—measured values, vertices of the broken lines—values calculated from the FFP attenuation characteristics; the lines themselves are guides for the eye only.

**Figure 5:**
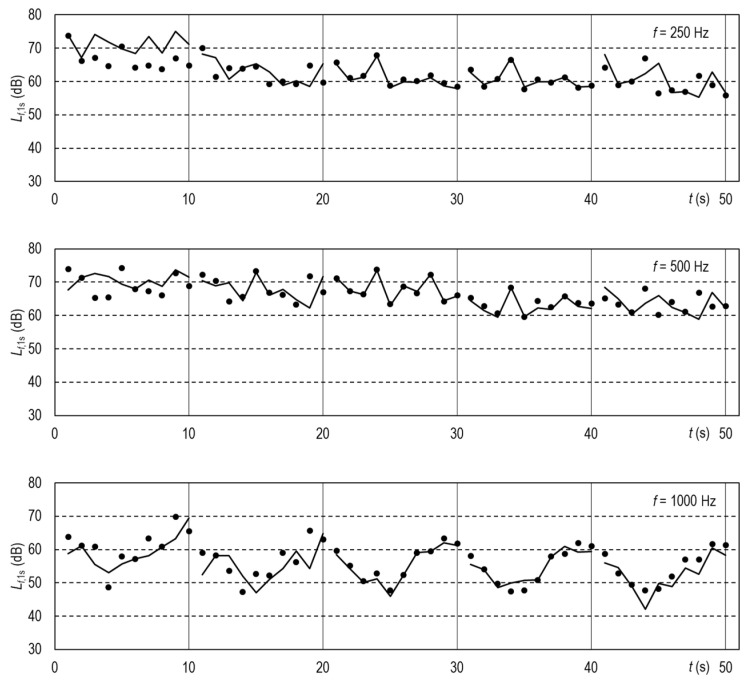

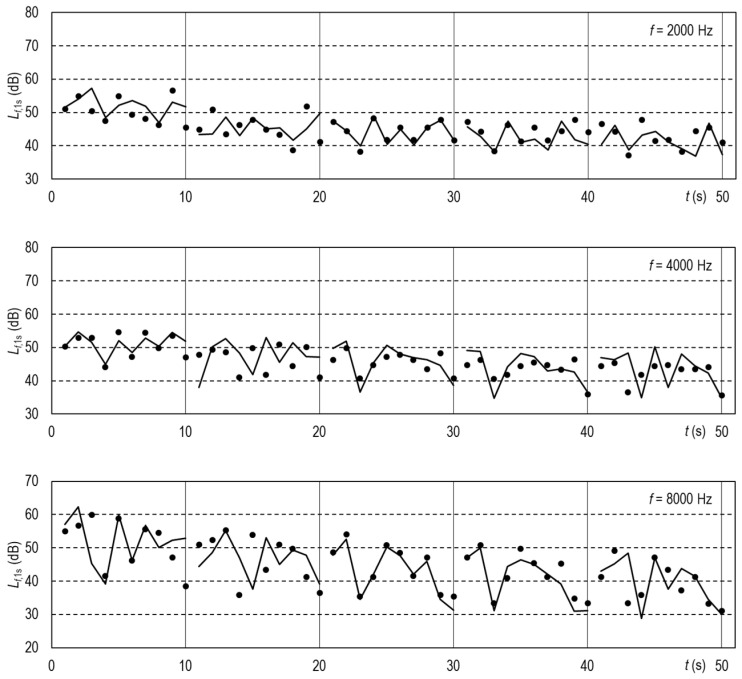
Time series of the sound pressure levels for the FFP3+ in octave bands averaged for 1 s-long intervals. A detailed explanation is in the caption of Figure 4.

*L_f_*_,1s_ (no FFP) in Equation (2) represents the sound pressure level measured for the uncovered TalkBox speaker. All of the time series are reported in five Supplementary Files “Speech_72dB_FFP”. Figure 4 and Figure 5 illustrate the results for FFP1 and FFP3+ and octave bands with center frequencies from 250 Hz to 8 kHz. A slight horizontal mismatch of the calculated and measured values may result from the sound recordings being not perfectly in phase. Examples of such mismatch are in the Supplementary Files.

## 4. Discussion

The white noise experiments showed that all the studied FFPs suppressed acoustic waves from the octave bands with center frequencies of 1 kHz and higher (Figure 3). Xue et al. [28] showed that the frequencies above 1 kHz in human speech are crucial for vowels articulation, thus for a proper understanding of the speech. According to French and Steinberg [29], four octave-wide bands with center frequencies from 1 to 8 kHz account for 20, 30, 25, and 5% of the perceived speech intelligibility (the numbers were calculated from the data reported by French and Steinberg in Table III of their paper and they are probably valid for non-tonal languages). Thus, the FFPs affected the speech transmission in the frequency range where 80% of the information is transferred, notably in the octave band with the center frequency of 2 kHz. As could have been expected, the better the filtration efficiency, the stronger the suppression. The exhalation valve mounted in the mask slightly increased the attenuation, particularly in the 16 kHz octave band. This frequency range is of no importance for speech intelligibility. It seems reasonable to suppose that suppression depends on the density and thickness of the mask material, such as the non-woven synthetics in the studied FFPs. Many such materials are in general use. For this reason, the reported attenuation characteristics can be inappropriate for other FFPs, even those of the same types. Thus, a generalization would be premature at this stage of the study.

**Figure 6:**
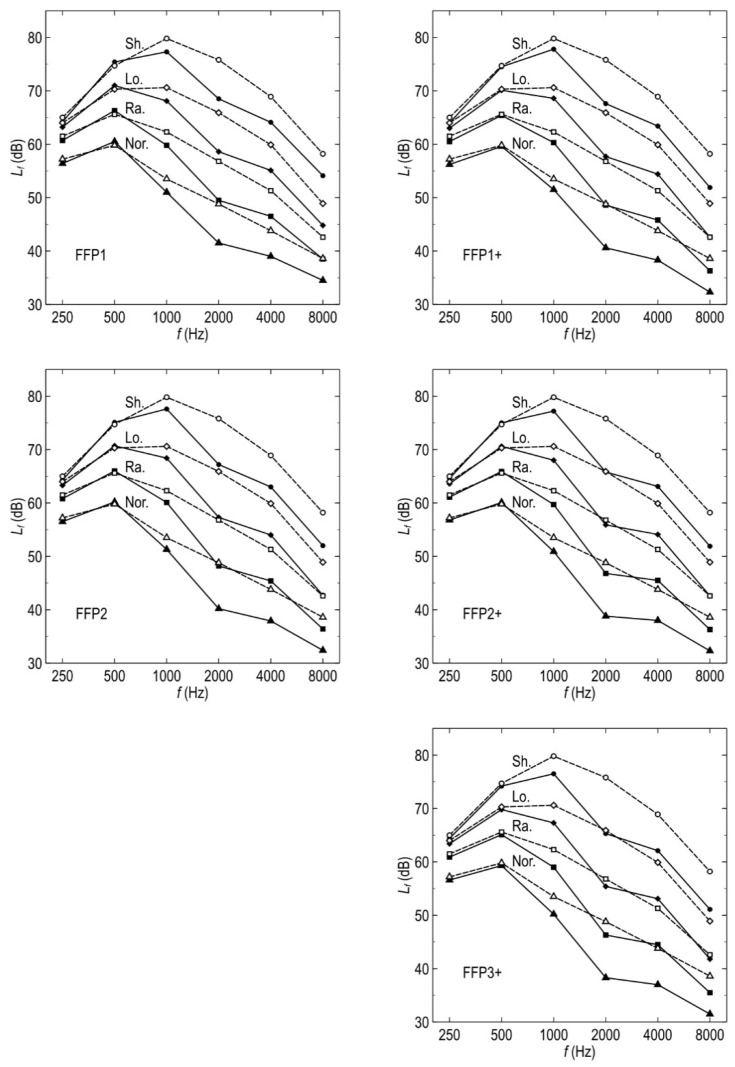
Disruptions caused by FFPs on the ANSI speech spectra. Empty symbols—ANSI spectra, filled symbols—spectra with the FFPs corrections: *L_f_*(ANSI)—Δ*L_f_*_,60s_. Lines are guides for the eye only. Speech levels: Nor.—normal, Ra.—raised, Lo.—loud, Sh.—shouted. Plus sign in the FFP symbol denotes a mask with an exhalation valve.

The simulated speech experiments evidenced that the time series of the speech calculated from the attenuation characteristics of the FFPs were very close to those measured directly (Figure 4 and Figure 5). Thus, human voice suppression can be analyzed semi-quantitatively based on a normalized speech spectrum, such as that reported in ANSI 3.5-1997 standard [27]. Figure 6 illustrates the disruptions caused by FFPs on the ANSI speech spectra expressed as the sound pressure levels at the one-meter distance in front of the speaker’s mouth. FFPs substantially decrease the speech loudness in the 2, 4, and 8 kHz-octave bands. To compensate for the change, the speaking person would have to raise the normal voice or even shout rather than speak loud where it is necessary. That would result in an increased share of low-frequency waves in the disrupted speech spectrum. Thus, FFPs not only attenuate the speech but change the timbre of voice. The latter is crucial for proper interpersonal communication. The changed voice timbre discloses the stress level and emotional arousal [30]. Non-verbal information in audible spectra influences emotional responses to speech. Disrupted speech could be particularly annoying for people with partial hearing loss [31]. As presbycusis impedes speech understanding [32], the attenuation of high-pitch tones by an FFP covering the speaker’s mouth could cause additional discomfort for elderly listeners. This ailment affects about one-third people of age between 65 and 74 years and almost half of those older than 75 [33]. Of course, louder speech requires more effort in inhaling the air, which causes additional discomfort for the mask wearer. Thus, an attenuation characteristic of masks would be a piece of welcome information for potential users.

## 5. Conclusions

1. All the studied FFPs suppress acoustic waves from the octave bands with center frequencies of 1 kHz and higher, i.e., in the frequency range responsible for 80% of the perceived speech intelligibility. In particular, FFPs significantly attenuate the acoustic waves belonging to the 2 kHz octave responsible for 30% of the intelligibility.
2. The better the mask filtration efficiency, the stronger is the sound suppression. The masks with exhalation valves suppress sound slightly more than their counterparts without such equipment; the difference is little except in the octave band with the center frequency of 16 kHz. The latter, however, has no practical importance for the understanding of speech.
3. The speaker–listener distance does not influence the characteristics of the speech deterioration significantly. To compensate for the FFP attenuation, a speaking person would have to raise the voice by one “loudness level” of the speech as defined in ANSI 3.5-1997 standard. Different attenuation in octave bands causes a change in the voice timbre. That can impede speech understanding.

The above conclusions suggest that the agencies for safety and health at work should consider including objective speech attenuation measurements in the relevant standards. Good communication is crucial for safety and comfort in the work environment.

## Figures and Tables

**Table 1:** Filtering half masks—research material, Reprinted/adapted with permission from Ref. [11].

Type/ Mark	Filtration Efficiency (%)	Recommended Application
FFP1	80	Construction, public works, heavy industry, healthcare
FFP2	94	Construction, public works, heavy industry, healthcare
FFP1+	94	Agriculture, horticulture, construction, public works, renovation, crafts, light industry, healthcare
FFP2+	99	Construction, public works, renovation works, crafts, light industry, healthcare
FFP3+	99	Construction, public works, renovation works, crafts, light industry, healthcare

**Table 2:** Attenuation of sound in octave bands with center frequency *f* by the five FFPs, Δ*L_f_*_,60s_, measured at two speaker–microphone distances and two white noise decibel levels, and the respective averaged values.

Mask	*f* (Hz)	Δ*L_f_*_,60s_ (dB)				
		1 m, 60 dB	1 m, 72 dB	5 m, 60 dB	5 m, 72 dB	Average
FFP1	125	0.7	0.4	0.2	0.1	0.4
	250	0.8	0.9	0.3	1.0	0.8
	500	−0.8	−0.9	−0.4	−0.9	−0.7
	1000	2.7	3.2	2.1	2.0	2.5
	2000	7.6	7.8	6.9	7.0	7.3
	4000	4.5	4.8	5.0	4.8	4.8
	8000	3.8	4.0	4.2	4.2	4.1
	16,000	5.9	5.8	5.2	5.8	5.7
FFP2	125	1.0	0.7	−0.1	0.0	0.4
	250	0.3	0.8	1.0	0.7	0.7
	500	−0.4	0.0	−0.4	−0.6	−0.4
	1000	2.5	2.6	1.6	2.1	2.2
	2000	9.2	9.1	7.9	8.3	8.6
	4000	5.8	6.4	5.7	5.6	5.9
	8000	6.1	6.6	6.1	6.0	6.2
	16,000	7.7	7.9	6.5	7.8	7.5
FFP1+	125	0.2	0.3	0.6	−0.1	0.3
	250	0.6	1.4	1.0	1.0	1.0
	500	0.5	0.3	0.1	−0.1	0.2
	1000	2.2	2.3	1.6	1.6	2.0
	2000	8.6	8.7	7.1	8.2	8.2
	4000	5.5	5.5	5.6	5.3	5.5
	8000	6.0	6.0	6.6	6.7	6.3
	16,000	10.9	11.0	8.2	10.1	10.2
FFP2+	125	0.6	0.4	0.7	−0.1	0.4
	250	−0.1	0.0	0.7	0.9	0.4
	500	−0.1	−0.1	−0.4	−0.7	−0.3
	1000	3.0	3.2	2.0	2.1	2.6
	2000	10.3	10.3	9.3	9.9	10.0
	4000	5.1	5.0	6.4	6.4	5.8
	8000	4.9	5.0	7.3	7.4	6.3
	16,000	11.5	11.9	8.8	11.3	11.0
FFP3+	125	1.3	1.6	1.6	1.0	1.4
	250	0.6	0.5	0.7	0.5	0.6
	500	0.6	0.4	0.4	0.5	0.5
	1000	3.6	3.7	2.9	2.8	3.3
	2000	10.7	10.7	9.9	10.4	10.5
	4000	6.5	6.6	7.1	7.1	6.8
	8000	6.5	6.5	7.3	7.9	7.1
	16,000	12.0	12.4	8.4	10.9	11.2

## Supplementary Materials

The following supporting information can be downloaded at: https://www.mdpi.com/article/10.3390/ijerph19127012/s1.

## References

[1] Setiawan F., Khazim I., Amri Z. (2018). Compliance of Personal Protective Equipment (PPE) N-Series Mask Type 8211 and the Description of Lung Function of the Coal Mining Workers, PT. X, Kutai Kartanegara, East Borneo. Adv. Sci. Lett..

[2] Teixeira R.L., Rezende R.N., Silva J.R.M.D., Paula L.E.D.R. (2018). Concentration and size of airborne particles in manufacturing environments. Rev. Árvore.

[3] Top Y., Adanur H., Öz M. (2016). Comparison of practices related to occupational health and safety in microscale wood-product enterprises. Saf. Sci..

[4] Ahmad I., Balkhyour M.A. (2020). Occupational exposure and respiratory health of workers at small scale industries. Saudi J. Biol. Sci..

[5] Majchrzycka K., Okrasa M., Szulc J., Gutarowska B. (2017). The impact of dust in filter materials of respiratory protective devices on the microorganisms viability. Int. J. Ind. Ergon..

[6] (2009). Respiratory Protective Devices—Filtering Half Masks to Protect against Particles—Requirements, Testing, Marking.

[7] Moreno-Casbas M.T. (2020). Factors related to SARS-CoV-2 infection in healthcare professionals in Spain. The SANICOVI project. Enferm. Clín..

[8] World Health Organization Coronavirus Disease (COVID-19): Masks, Updated 5 January 2022. https://www.who.int/emergencies/diseases/novel-coronavirus-2019/question-and-answers-hub/q-a-detail/coronavirus-disease-covid-19-masks.

[9] Neuwirth M., Mattner F., Otchwemah R. (2020). Adherence to personal protective equipment use among healthcare workers caring for confirmed COVID-19 and alleged non-COVID-19 patients. Antimicrob. Resist. Infect. Control..

[10] Cheberyachko S., Cheberyachko Y., Naumov M. Use of dust masks at coal enterprises. Proceedings of the School of Underground Mining Location: Technical and Geoinformational Systems in Mining.

[11] www.deltaplus.eu.

[12] Bertoli S., Leone A., De Amicis R., Foppiani A., Osio D., Battezzati A. (2021). Effects of wearing a FFP2 mask on indirect calorimetry measurements: A pilot study. Clin. Nutr. ESPEN.

[13] Choi S., Park R., Hur N., Kim W. (2020). Evaluation of wearing comfort of dust masks. PLoS ONE.

[14] Brotto D., Sorrentino F., Agostinelli A., Lovo E., Montino S., Trevisi P., Favaretto N., Bovo R., Martini A. (2021). How great is the negative impact of masking and social distancing and how can we enhance communication skills in the elderly people?. Aging Clin. Exp. Res..

[15] Cohn M., Pycha A., Zellou G. (2021). Intelligibility of face-masked speech depends on speaking style: Comparing casual, clear, and emotional speech. Cognition.

[16] Caniato M., Marzi A., Gasparella A. (2021). How much COVID-19 face protections influence speech intelligibility in classrooms?. Appl. Acoust..

[17] Bandaru S., Augustine A., Lepcha A., Sebastian S., Gowri M., Philip A., Mammen M. (2020). The effects of N95 mask and face shield on speech perception among healthcare workers in the coronavirus disease 2019 pandemic scenario. J. Laryngol. Otol..

[18] Oren L., Rollins M., Gutmark E., Howell R. (2021). How face masks affect acoustic and auditory perceptual characteristics of the singing voice. J. Voice.

[19] de Araújo Andrade T., Nascimento Junior J.A.C., Santos A.M., Borges L.P., Quintans-Junior L.J., Walker C.I.B., Frank L.A., Serafini M.R. (2020). Technological Scenario for Masks in Patent Database During COVID-19 Pandemic. AAPS PharmSciTech.

[20] Cheberiachko S.I., Cheberiachko Y.I., Shaikhlislamova I.A. (2020). Designing of Half-Masks of Filtering Respirators. Sci. Innov..

[21] Gök K., Selçuk A.B., Gök A. (2021). Computer-Aided Simulation Using Finite Element Analysis of Protect Against to Coronavirus (COVID-19) of Custom-Made New Mask Design. Trans. Indian Inst. Met..

[22] Cheberiachko S.I., Cheberiachko Y.I., Deriuhin O.V., Slavinskyi D.V. (2019). Dust Mask with a Pressure Drop Measuring Device. Visnyk NTUU KPI Seriia—Radiotekhnika Radioaparatobuduvannia.

[23] (2013). Electroacoustics—Sound Level Meters—Part 1: Specifications.

[24] (2017). Electroacoustics—Sound Calibrators.

[25] Nowak E. (2000). Atlas Antropometryczny Populacji Polskiej: Dane do Projektowania.

[26] (2020). Sound System Equipment—Part 16: Objective Rating of Speech Intelligibility by Speech Transmission Index.

[27] (1997). Methods for Calculation of the Speech Intelligibility Index.

[28] Xue Y., Marxen M., Akagi M., Birkholz P. (2021). Acoustic and articulatory analysis and synthesis of shouted vowels. Comput. Speech Lang..

[29] French N.R., Steinberg J.C. (1947). Factors governing the intelligibility of speech sounds. J. Acoust. Soc. Am..

[30] Kiese-Himmel C. (2021). The frequency of the voice in the conversation. Speech-Voice-Hearing.

[31] Buono G.H., Crukley J., Hornsby B.W.H., Picou E.M. (2021). Loss of high- or low-frequency audibility can partially explain effects of hearing loss on emotional responses to non-speech sounds. Hear. Res..

[32] Vogelzang M., Thiel C.M., Rosemann S., Rieger J.W., Ruigendijk E. (2021). Effects of age-related hearing loss and hearing aid experience on sentence processing. Sci. Rep..

[33] Hearing Loss: A Common Problem for Older Adults, NIH—National Institute on Aging, U.S. Department of Health & Human Services. https://www.nia.nih.gov/health/hearing-loss-common-problem-older-adults.

